# Gel Immersion Endoscopic Mucosal Resection (EMR) for Superficial Nonampullary Duodenal Epithelial Tumors May Reduce Procedure Time Compared with Underwater EMR (with Video)

**DOI:** 10.1155/2022/2040792

**Published:** 2022-06-15

**Authors:** Takeshi Yamashina, Masaaki Shimatani, Yu Takahashi, Masahiro Takeo, Natsuko Saito, Hironao Matsumoto, Takeshi Kasai, Masataka Kano, Kimi Sumimoto, Toshiyuki Mitsuyama, Hiroyuki Marusawa, Akiyoshi Nishio, Takafumi Yuba, Toshihito Seki, Makoto Naganuma

**Affiliations:** ^1^Division of Gastroenterology and Hepatology, Kansai Medical University Medical Center, Moriguchi, Osaka, Japan; ^2^Department of Gastroenterology and Hepatology, Osaka Red Cross Hospital, Osaka, Japan; ^3^The Third Department of Internal Medicine, Kansai Medical University, Hirakata, Osaka, Japan; ^4^Division of Liver Disease Center, Kansai Medical University Medical Center, Moriguchi, Osaka, Japan

## Abstract

**Materials and Methods:**

This was a retrospective cohort study conducted in two municipal hospitals. We identified 24 patients with SNADETs of 3–18 mm in diameter who underwent UEMR or GIEMR. One lesion was excluded from the analysis because it was found to be in the stomach after surgery. The primary outcome was procedure time.

**Results:**

GIEMR significantly reduced the procedure time compared with UEMR (5 min vs. 10 min, *P* = 0.016). There was no significant difference between the UEMR and GIEMR groups for *en bloc* resection rate (93% vs. 100%, *P* = 1.0) and R0 resection rate (57% vs. 80%, *P* = 0.39). No serious complications were observed in either group.

**Conclusions:**

GIEMR of SNADET has the potential to reduce procedure time compared with UEMR and may be particularly effective in areas where immersion in water is difficult.

## 1. Introduction

Recent advances in endoscopic imaging techniques have led to an increase in the detection of superficial nonampullary duodenal epithelial tumors (SNADETs) [1]. Duodenal adenomas can develop into cancer [2]; therefore, early treatment is expected to improve the prognosis. However, there are still no clear criteria for choosing an endoscopic treatment strategy for SNADETs. Endoscopic mucosal resection (EMR) is the most common endoscopic treatment method for SNADETs, but it is problematic because of serious adverse events [3] and low curative outcomes leading to a high recurrence rate [4–6]. Underwater EMR (UEMR) was reported by Binmoeller et al. [7] and has recently attracted attention as a safe and effective treatment method for SNADETs [8–10]. However, water flow means that it is difficult to accumulate water in some areas. Additionally, water tends to mix with other substances, including intestinal fluids and residues, resulting in poor visibility. In such cases, it is sometimes difficult to perform UEMR because it requires a large amount of water and takes a long time. In 2021 a new endoscopic procedure and treatment gel product (VISCOCLEAR®; Otsuka Pharmaceuticals Factory, Tokushima, Japan) was launched in Japan and reported to be useful for identifying active sources of gastrointestinal hemorrhage [11–13]. Unlike water, this gel product does not mix with intestinal fluids or blood, thus ensuring a good view of the gastrointestinal tract. Furthermore, it tends to stay in the injected area due to its viscoelasticity, which may shorten the treatment time. Previously, we reported the efficacy and safety of gel immersion EMR (GIEMR), also called under-gel EMR, with which SNADETs were immersed in this gel instead of water and then resected [14]. In the present study, we compared the efficacy and safety of UEMR and GIEMR for treatment of SNADETs.

## 2. Materials and Methods

### 2.1. Patients

This retrospective cohort study was performed in two municipal hospitals, Kansai Medical University Medical Center and Osaka Red Cross Hospital. The study protocol was approved by the Ethics Committees of Kansai Medical University Medical Center (No. 2021304) and Osaka Red Cross Hospital (No. J-0188). All patients were informed of the study and provided informed consent. This study was also carried out in accordance with the Declaration of Helsinki. We enrolled patients with SNADETs less than 20 mm in diameter who underwent UEMR or GIEMR between January 2018 and December 2021 by the indications for endoscopic treatment in the guidelines [15]. The criteria for UEMR or GIEMR were patients with adenoma or more advanced lesions by preoperative endoscopic biopsy. These patients were identified consecutively in prospectively maintained databases at the two hospitals. We included patients referred for endoscopic resection of SNADETs and all polyps resected for SNADETs found during screening at our institutions. This manuscript was written in accordance with the Strengthening the Reporting of Observational Studies in Epidemiology (STROBE) statement [16].

During the study period, 24 SNADETs in 23 patients were treated at the Osaka Red Cross Hospital or Kansai Medical University Medical Center. One lesion was excluded from the analysis because it was found to be in the stomach after surgery. The final analysis included 23 patients and 24 lesions; 14 were in the UEMR group, and 10 in the GIEMR group.

### 2.2. EMR Procedures

The GIEMR procedure included the following: (1) the duodenal lumen was fully deflated; (2) a 50 ml syringe filled with gel (VISCOCLEAR®; Otsuka Pharmaceuticals Factory, Tokushima, Japan) was attached to the BioShield irrigator (U.S. Endoscopy, Mentor, OH, USA), gel was injected through the accessory channel of the endoscope (GIF-Q260J; Olympus Medical Systems, Tokyo, Japan), and the lesion was completely immersed in the gel; (3) snaring the lesion and the surrounding mucosa with a bipolar snare (DRAGONARE™ 26 mm; Xemex, Tokyo, Japan); and (4) the lesion was resected using a high-frequency electrical generator (VIO® 300 D; Erbe Elektromedizin, Tübingen, Germany) with the following settings: auto-cut mode, effect 3, 30 W; forced coagulation mode, effect 1, 15 W.

The UEMR procedure included the following: (1) the duodenal lumen was fully deflated; (2) normal saline was injected into the lumen via the water jet channel of the endoscope (GIF-Q260J; Olympus Medical Systems) with a mechanical water pump (OFP-2; Olympus Medical Systems), and the lesion was completely immersed; (3) snaring the lesion and the surrounding mucosa with a bipolar snare (DRAGONARE™ 26 mm; Xemex, Tokyo, Japan) which has recently been used with the expectation of reducing the damage to the muscle layer caused by the electric current and heat [17, 18], or a monopolar snare (SnareMaster 15 mm; Olympus Medical System, Tokyo, Japan); and (4) the lesion was resected using a high-frequency electrical generator (VIO® 300 D; Erbe Elektromedizin) with the following settings: autocut mode, effect 3, 30 W; forced coagulation mode, effect 1, 15 W (bipolar snare) or Endo cut Q mode, effect 2, duration 1, interval 6, forced coagulation effect 2, 20 W (monopolar snare).

Finally, in both methods, closure of the mucosal defect was performed using an EZ Clip (Olympus Medical Systems).

### 2.3. Outcomes

The primary endpoint in this study was the difference in procedure time between the GIEMR and UEMR groups. The procedure time was defined as the time from the start of normal saline or gel injection into the lumen until the polyp was completely removed. *En bloc* resection rate, R0 resection rate, and adverse events were evaluated as secondary endpoints. *En bloc* resection was defined as endoscopically evaluated removal of the lesion in one piece, and R0 resection was when the horizontal and vertical margins were also negative histopathologically. Perforation was defined as visible luminal contents outside the gastrointestinal tract through the hole, or free or retroperitoneal air on abdominal computed tomography after endoscopic resection. Intraoperative bleeding was defined as bleeding during or immediately after endoscopic resection that required an endoscopic hemostatic procedure. Delayed bleeding was defined as hematochezia occurring >24 h after endoscopic resection that required an endoscopic hemostatic procedure.

### 2.4. Statistical Analysis

Categorical data were analyzed using Fisher's exact test or *χ*^2^ test. Quantitative data were compared using Student's *t* test or Mann–Whitney *U* test. *P* < 0.05 (two-sided) was considered significant. SPSS Statistics version 23 (Chicago, IL, USA) was used for all statistical analyses.

## 3. Results

### 3.1. Baseline Data

The 24 lesions in 23 patients were divided into the UEMR (*n* = 14) and GIEMR (*n* = 10) groups. Before November 2020, we only performed UEMR and after November 2020, when VISCOCLEAR was launched, we only performed GIEMR. The procedures were performed by 2 expert (Certification from Japanese Gastroenterological Endoscopy Society) and 2 nonexpert endoscopists. The number of experiences of gastrointestinal endoscopic treatment before this study for two expert endoscopists was EMR ≥ 100 and UEMR ≥ 10 and, for two nonexpert endoscopist, was EMR ≥ 10 and UEMR = 0. The baseline characteristics are presented in Table 1. There were 15 men and eight women with a median age (range) of 66 (41–81) years. Three lesions (12.5%) were located in the bulbs and the other 21 in the second portion of the duodenum. The median tumor diameter size (range) was 9 (3–18) mm. There were no differences in age, gender, location, morphology, and histological type between the UEMR and GIEMR groups, although the GIEMR group had a significantly larger median tumor diameter size (6 mm vs. 10 mm, *P* = 0.0077).

### 3.2. Procedure-Related Outcomes

The procedure-related outcomes are presented in Table 2. The median procedure time was significantly shorter in the GIEMR group than in the UEMR group (10 min vs. 5 min, *P* = 0.016), with Cohen's *d* revealing large effect size (0.99). There was no significant difference between the UEMR and GIEMR groups in *en bloc* resection rate (93% vs. 100%, *P* = 1.0) and R0 resection rate (57% vs. 80%, *P* = 0.39), with Cramer's *V* revealing small effect size (0.18 and 0.24, respectively). In the non-R0 resection cases, three cases had positive horizontal margins and three cases were unclear in the UEMR group. In the GIEMR group, two cases had unclear horizontal margins. The median quantity of water or gel required to immerse the lesion was significantly less in the GIEMR group (170 ml vs. 100 ml, *P* = 0.0012), with Cohen's *d* revealing large effect size (1.49), although this was not recorded in half of the UEMR group. The mucosal defects after endoscopic resection were successfully closed with endoscopic clips in all cases.

### 3.3. Adverse Events

There were no cases of intraoperative or delayed perforation and delayed bleeding in either group. There was one case of intraoperative bleeding in the GIEMR group and one in the UEMR group; both of which were controlled with endoscopic clips (Table 2). No other serious complications were observed in either group.

## 4. Discussion

To the best of our knowledge, this is the first trial to compare the efficacy and safety of UEMR and GIEMR. In this study, GIEMR had a significantly shorter procedure time than UEMR had, without increasing the incidence of adverse events. Furthermore, despite the GIEMR group having a significantly larger median tumor diameter size, GIEMR had higher *en bloc* and R0 resection rates than UEMR had, although the difference was not significant. Furthermore, an inexperienced endoscopists were able to perform GIEMR easily in two cases. The results indicate that GIEMR may be useful in daily clinical practice.

Gel immersion endoscopy was first reported by Yano et al. as a new treatment for gastrointestinal bleeding [11]. Water quickly mixes with blood, intestinal fluids, and food residues, making it difficult to maintain a clear field of view and this leads to difficulties with hemostasis. Gel immersion allows a clear space to be created in front of the endoscope to ensure a good field of view by injecting a gel instead of water through the attached channel of the endoscope. This makes it easier to identify the source of bleeding and helps to ensure endoscopic hemostasis. It is also reported to be useful for gastrointestinal bleeding at various sites [13]. In addition, there are several case reports, including ours, on the application of this gel for endoscopic submucosal dissection and EMR [14, 19–22]. However, this is believed to be the first trial to compare the efficacy and safety of UEMR and GIEMR.

**Figure 1:**
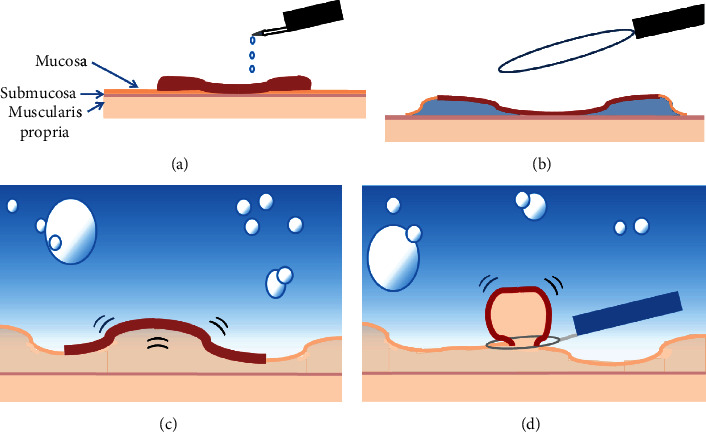
(a, b) SNADETs are likely to cause fibrosis by biopsy, making it difficult to obtain good lifting by submucosal injection. In addition, the injected fluid tends to spread horizontally due to the coarse submucosal layer which makes snaring more difficult. (c, d) Water immersion decreased the luminal extension force and increased mucosal and submucosal buoyancy, which facilitates their snaring.

Although EMR is a common endoscopic treatment for SNADETs up to 20 mm, the adverse event rates are reported to be 0%–3% for intraoperative perforation, 0%–3.6% for delayed perforation, and 0%–13% for delayed bleeding [3, 23–25]. In particular, delayed perforation has a serious course, and EMR is never a safe treatment. There are several reasons why accidental injuries are not uncommon in duodenal EMR. The lumen of the duodenum is tightly curved at the inferior and superior duodenal angles and is susceptible to extension and flexure, which often makes it difficult to manipulate. Additionally, the mucosa and submucosa of the duodenum are thin and can be easily perforated, or even a small biopsy fragment can cause severe fibrosis. And the injected fluid tends to spread horizontally due to the coarse submucosal layer (Figures 1(a) and 1(b)). These difficulties in snaring lead to piecemeal resection, and the resultant repeated electrocautery may be one of the factors causing adverse events [3].

UEMR has attracted much attention in recent years and is reported to be a more effective and safer endoscopic procedure than conventional EMR [26–28]. Compared to conventional EMR, in UEMR, the mucosal deflexion caused by deflation and water immersion makes the lesion more polypoid and the lumen is straighter, which facilitates snaring and leads to higher *en bloc* and R0 resection rates [7, 29](Figures 1(c) and 1(d)). The lack of injection also allows the ulcer edges after endoscopic resection to shrink and soften, which facilitates clip closure after resection. In addition to these factors, the heat sink effect of immersion in water, which reduces thermal injury, is thought to prevent adverse events [30]. The favorable treatment outcomes of GIEMR in the present study suggest that these benefits can be equally obtained in gel immersion just like UEMR.

**Figure 2:**
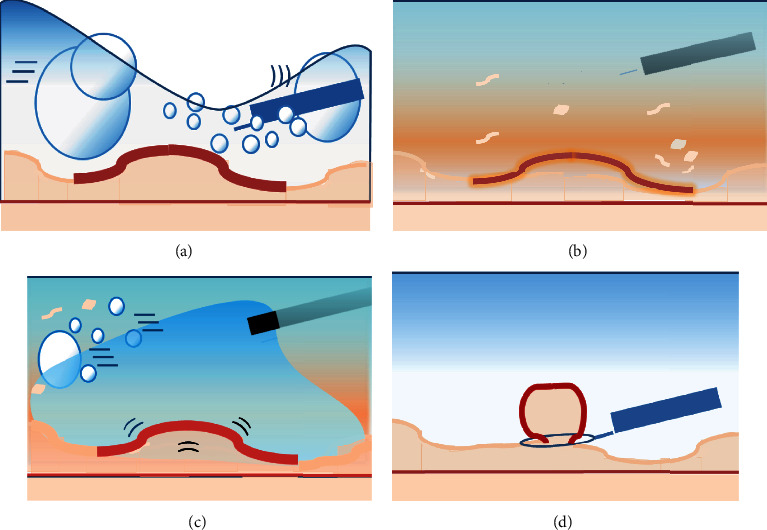
(a, b) Underwater immersion, the endoscopic view is sometimes poor due to the difficult accumulation of water and mixture of air bubbles or intestinal fluids. (c, d) The viscoelasticity of the gel allows it to stay in the lumen and easily remove air bubbles and intestinal fluids without mixing, resulting in a good field of view and facilitating snaring.

**Figure 3:**
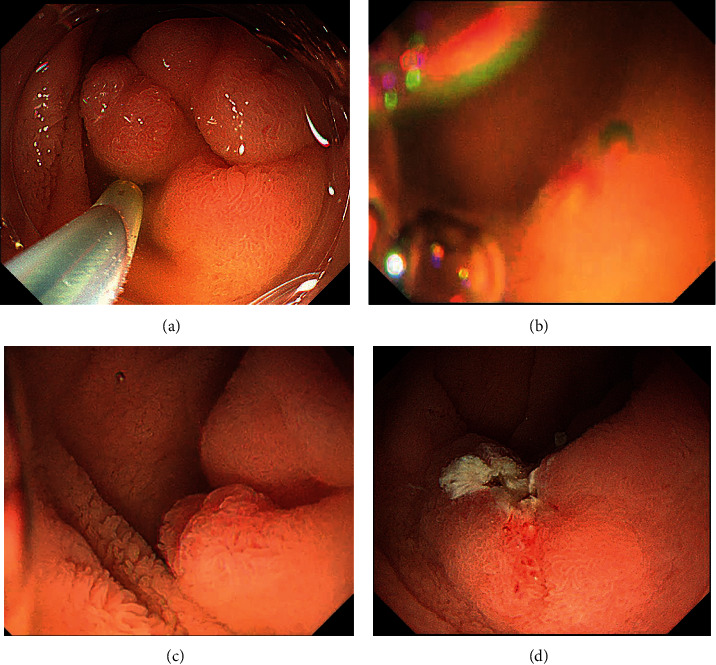
(a) SNADETs in the superior duodenal angle. (b) Air and bile cause poor visibility in underwater. (c) The viscoelasticity of the gel allows it to stay in the lumen and easily remove air bubbles and intestinal fluids without mixing, resulting in a good field of view. (d) The SNADETs resected *en bloc* resection without any adverse events.

However, immersion in water may cause mixing with intestinal fluids such as bile, and air bubbles may flow in, resulting in poor visibility (Figures 2(a), 2(b), 3(a), and 3(b)). Removal might require a lot of water or take a long time to achieve. In this study, GIEMR was able to shorten the procedure time compared with that of UEMR because a good field of view was easily obtained with the use of gel (Figures 2(c), 2(d), 3(c), and 3(d)) (Video 1). Even if it is difficult to immerse the lesion in water because of air bubbles or water outflow, it may still be possible to perform UEMR by repeating several times the process of air deflation and injection of large volumes of water. However, repeating this process is time consuming and may cause peristalsis that makes resection more difficult. Although it is not directly comparable due to different backgrounds and definition, previous reports have shown that the procedure time for SNADETs in UEMR is 5–11 min [8, 9, 26]. The median treatment time for GIEMR in the present study was 5 min, which was shorter than in previous studies. This is because the viscoelasticity of the gel allows the air bubbles to be easily removed and allows the gel to remain in the lumen, so a small amount is sufficient. This is supported by the fact that the median amount of gel required to immerse the lesion was only 100 ml, which was significantly less than the amount of water required in UEMR in the present study and less than the 330 ml required in the previously reported UEMR study [9]. A five-minute reduction in procedure time may be short in daily clinical practice; however, the extended accumulation of water in the duodenal tract can cause peristalsis and discomfort for the patient. Furthermore, adverse events for duodenal endoscopic procedures are not uncommon and serious; hence, we believe that a reduction in procedure time is meaningful.

In our study, GIEMR achieved good endoscopic outcomes for SNADETs, with 100% *en bloc* resection rate and 80% R0 resection rate without complications. It has been reported that the *en bloc* resection rate decreases when the diameter of the lesion exceeds 10 mm [31, 32]. However, in the GIEMR group in our study, although the diameter of lesions exceeded 10 mm in eight cases, *en bloc* resection was obtained in all cases. Previous studies have reported *en bloc* resection rates of 75.4%–100% and R0 resection rates of 50.8%–88.2% for SNADET in UEMR [8–10, 26–28]. Our study had a small number of cases; however, both the *en bloc* and R0 resection rates of GIEMR were higher compared with those in previous UEMR studies.

There were several limitations to our study. First, detailed patient data were collected from medical records retrospectively. We routinely recorded endoscopic records and symptoms for all patients undergoing UEMR or GIEMR according to their care plan. The possibility cannot be ruled out; however, that data on some patients' symptoms may have been missing from some records. Second, this study in a small, limited number of patients was conducted in two city hospitals. Standardized protocols and perioperative management provide pure results and large effect size supports our conclusion but do not allow for evaluation of outcomes at other institutions because of the possible influence of various confounding factors, such as differences in injection methods, endoscopists' experience, and patient background. Further multicenter randomized studies will provide more detailed information on GIEMR.

## 5. Conclusion

GIEMR was safe and had good therapeutic results for SNADETs and showed the potential to accelerate the procedure time compared with UEMR. This method may be particularly useful in areas where immersion in water is difficult. A multicenter study is needed to confirm the validity of our results.

## Figures and Tables

**Table 1:** Characteristics of patients and lesions.

	UEMR	GIEMR	*P*
Patients/lesions	14/14	9/10	0.66^1^
Male	10 (71%)	5 (56%)	
Female	4 (29%)	4 (44%)	
Median age (range, years)	62 (41–78)	67 (51–81)	0.16^2^
Location			0.64^3^
Bulb	1 (7%)	2 (20%)	
Second portion, preampulla	5 (36%)	3 (30%)	
Second portion, postampulla	8 (57%)	5 (50%)	
Morphology			0.30^3^
Sessile type	1 (7%)	3 (30%)	
Superficial elevated type	10 (71%)	6 (60%)	
Superficial depressed type	3 (21%)	1 (10%)	
Median tumor diameter size (range, mm)	6 (3–12)	10 (7–18)	0.0077^2^
Histological type			0.10^3^
Adenoma	11 (79%)	5 (50%)	
Intramucosal carcinoma	3 (21%)	5 (50%)	
Operators' experience			0.16^1^
Expert	14 (100%)	8 (80%)	
Nonexpert	0 (0%)	2 (20%)	

^1^Fisher's exact test. ^2^Mann–Whitney *U* test. ^3^*χ*^2^ test. GIEMR: gel immersion endoscopic mucosal resection; UEMR: underwater endoscopic mucosal resection.

**Table 2:** Procedure-related outcomes.

	UEMR	GIEMR	Effect size	*P*
*En bloc* resection	13/14 (93%)	10/10 (100%)	0.18^1^	1.0^1^
R0 resection	8/14 (57%)	8/10 (80%)	0.24^1^	0.39^3^
Median procedure time (range, minutes)	10 (3–25)	5 (3–10)	0.99^2^	0.016^4^
Median amount of filling water/gel (range, ml)	170^5^ (100–480)	100 (50–100)	1.49^2^	0.0012^4^
Adverse events			NaN	1.0^3^
Intraprocedural or delayed perforation	0	0		
Intraoperative bleeding	1	1		
Delayed bleeding	0	0		

^1^Cramer's *V*. ^2^Cohen's *d*. ^3^Fisher's exact test. ^4^Mann–Whitney *U* test. ^5^Only seven recorded cases. GIEMR: gel immersion endoscopic mucosal resection; UEMR: underwater endoscopic mucosal resection.].

## Data Availability

The data used to support the findings of this study are available from the corresponding author upon request.

## Supplementary Materials

Supplementary Materials Video legend A 7 mm lesion in the superior duodenal angle was successfully resected by converting from UEMR to gel immersion EMR.
