# Supplementary material for: Gel Immersion Endoscopic Mucosal Resection (EMR) for Superficial Nonampullary Duodenal Epithelial Tumors May Reduce Procedure Time Compared with Underwater EMR (with Video)
Source: Gastroenterol Res Pract. 2022 Jun 15;2022:2040792. doi: 10.1155/2022/2040792 (PMC9217606; doi:10.1155/2022/2040792)
Supplement: Supplementary Materials — Video legend A 7 mm lesion in the superior duodenal angle was successfully resected by converting from UEMR to gel immersion EMR. [file 2040792.f1.docx]

Video download

<https://drive.google.com/file/d/1Gf9CbIcX-RSupoze5iaglWfEKZJ1NGFe/view?usp=sharing>
